# Challenges and lessons learned in re-filming the WHO mhGAP training videos for Sri Lankan context - a qualitative study

**DOI:** 10.1186/s13031-020-00259-z

**Published:** 2020-02-13

**Authors:** Shannon Doherty, Giselle Dass, Anne Edward, Gergana Manolova, Madonna Solomon

**Affiliations:** 1 Faculty of Health, Education, Medicine, and Social Care, Anglia Ruskin University, Bishop Hall Lane, Chelmsford, CM1 1SQ UK; 2 THEME Institute, Colombo, Sri Lanka; 3 Bristol Medical School, University of Bristol, Bristol, UK; 4 Globally Minded Foundation, Institute of Psychiatry, Psychology and Neuroscience, King’s College London, London, UK

**Keywords:** Sri Lanka, mhGAP, Adaptation, Video production, Qualitative study

## Abstract

**Background:**

Understanding and addressing the unmet mental health needs burden in the Northern Province of Sri Lanka is the subject of the COMGAP-S two-phase study. Phase Two involves the implementation of the World Health Organization’s mental health Gap Action Programme (mhGAP) in primary healthcare settings. As part of the contextual adaptation of mhGAP, eleven of the videos provided in the mhGAP training package have been re-filmed by a local team. We investigated the challenges, barriers and good practices of this adaptation effort from the point of view of team participants.

**Methods:**

Twelve persons from the adaptation team, including students of medicine and drama, doctors, drama lecturers and professionals, consented to in-depth individual interviews following an open-ended topic guide with a member of the COMGAP-S study team. Interviews were recorded, transcribed, translated as necessary, and subjected to thematic analysis.

**Results:**

The majority of participants perceived the process positively and had pride in their involvement. Expectations, opportunities, and exposure were discussed as stemming from the video production. The main challenges derived from the analysis were lack of discussion around budgeting, logistical difficulties, struggles with team cooperation, and creative differences. Issues around exact translation into the local Tamil dialect and modelling around mental health were emphasised by the majority of participants. Potential uses for the videos were identified beyond the current study and recommendations included setting out clear guidance around available funding and role allocation, and increasing the flexibility in adapting the material.

**Conclusions:**

This study illustrated details of the adaptation of existing video materials to facilitate locally-based training for non-specialists on mhGAP curricula. With this, we have added to the knowledge base on conducting cultural and language adaptations and our findings indicate participants felt adapting the mhGAP films to local context was vital to ensuring training materials were culturally appropriate and valid.

**Trial registration:**

This project was nested within the larger COMGAP-S clinical trial. Ethics approval was granted from the Ethics Review Committee, Faculty of Medicine, University of Jaffna (J/ERC/17/81/NDR/0170) and the Faculty Research Ethics Panel, Faculty of Medical Science, Anglia Ruskin University (SC/jc/FMSFREP/16/17076). The project is registered with the Sri Lankan Clinical Trial Registry (SLCTR/2018/008) and listed on the ISRCTN registry (trial ID ISRCTN62598070).

## Background

During a protracted civil conflict (1983–2009) in Sri Lanka, approximately 100,000 people were displaced, and hundreds of thousands were injured [1, 2]. The 2004 tsunami then further contributed to substantial internal displacement within the country [3]. Since the end of conflict in 2009, internally displaced people (IDPs) in Sri Lanka have been returning to areas of origin.

The prevalence of mental health disorders associated with the experience of conflict varies across countries and cultures [4–6]. In low-resource settings in particular, such as in the post-conflict regions of Sri Lanka, consequences of mental health disorders can be compounded by lack of access to, and lack of integration of mental health services into primary care systems.

Sri Lanka has a well-established primary care system, however the treatment gap for those seeking mental health services is still substantial. To address this, a five-year study entitled, “Integrating mental health into primary care for post-conflict populations in Northern Sri Lanka” (COMGAP-S), funded by the Centers for Disease Control and Prevention (CDC) USA was implemented. The first phase of COMGAP-S, an epidemiological survey to investigate the unmet mental health need at primary care level within the post-conflict region of Northern Province, Sri Lanka was completed in 2016. Results found anxiety prevalence was reported at 46.7% (95% CI, 41.9–51.5), depression at 41.1% (95% CI, 38.7–44.5), expression of somatoform symptoms at 27.6% (95% CI, 23.6–31.5), psychosis with hypomania at 17.6% (95% CI, 13.3–21.9), and post-traumatic stress disorder at 13.7% (95% CI, 10.6–16.8) [7]. These findings indicate a substantial unmet need for mental health services at primary care level in the region. Evidence indicates that treatment of common mental disorders at primary care level can be effective and, along with early intervention, can contribute to the reduction of global burden of disease [8].

Due to the high unmet mental health needs of those attending primary care in post-conflict Northern Province and a lack of specialised care in the region, Phase 2 of COMGAP-S aims to train primary care practitioners to identify, treat, and manage patients with mental health disorders. The training programme utilised in Phase 2 is the World Health Organisation’s (WHO) Mental Health GAP Action Program (mhGAP) training package. The Mental Health Intervention Guide 2.0 and 25 training videos form part of the package. The mhGAP training videos were funded and shot by the International Medical Corps (IMC) for the WHO and are freely available online (https://www.youtube.com/user/mhGAPtraining). These videos were originally filmed in Arabic language and are subtitled in English and French.

According to White and Sashidharan one of the limitations of mhGAP programmes is the lack of emphasis placed on the importance of inclusion of cultural and social factors within local contexts [9]. While the WHO does recommend that mhGAP be culturally adapted for the setting, this is left to the discretion of the implementing research teams. Research suggests the mhGAP programme may require further consultation with local stakeholders, particularly service users, to ensure it is culturally appropriate [9]. Some research studies on its implementation have illustrated and reflected on adaptations made, for example on the change of content to better fit the epidemiology of the setting or on translation and rearrangement of the chapter flow [10, 11]. In all settings the input from local practitioners and stakeholders is considered essential to the process of adaptation to ensure best fit with local expressions of mental distress, cultural practices, and most frequently encountered issues in clinical practice.

Due to successful previous pilot work in Northern Province with primary care practitioners [12], a number of key challenges and examples of best practice were already known to the current project team. Lessons learned from the Siriwardhana et al. pilot study were derived from qualitative research with participating medical professionals, who underscored the need for locally relevant training materials delivered in the local language [13]. As noted above, while the mhGAP package does include a set of freely available training videos, participants in the Siriwardhana et al. study concluded that provision of videos in the local Tamil language would be more appropriate and effective. Other researchers have also found that videos included in the mhGAP package were not necessarily suitable due to issues of language and culture, and instead role-play was prioritised to enhance the learning process [14].

In light of this, the COMGAP-S study team decided to re-film the mhGAP training videos within the Sri Lankan context and in the Tamil language. This video production was completed in partnership between Anglia Ruskin University (UK), the THEME Institute and the University of Jaffna (Sri Lanka). The following 11 videos were re-filmed in Sri Lankan context, in the Tamil language dialect spoken in the Northern Province of Sri Lanka, with English subtitles: depression assessment, depression management, depression follow-up, alcohol use, medically unexplained somatic symptoms, psychosis assessment, psychosis management, deliberate self-harm, behavioural disorders assessment, behavioural disorder management, developmental disorders. These particular videos were re-filmed based on needs articulated through the Phase 1 results of COMGAP-S [7]. Videos were filmed in various locations reflective of local context such as hospitals and outpatient clinics.

In order to understand the process, challenges, and strengths involved in re-filming the mhGAP videos for the Sri Lankan context, a series of interviews were undertaken with participants to understand their experience. Our hope is that this study will provide future researchers with greater understanding of the complexities and best practice strategies associated with locally adapting WHOmhGAP video material.

## Methods

### Study design and setting

This was a qualitative case study. In-depth interviews were utilised to explore the experiences of those involved in the cultural adaptation and filming of the WHO mhGAP videos for the Sri Lankan context. Interviews were conducted within the Northern Province of Sri Lanka at the University of Jaffna by trained researchers from the THEME Institute.

### Sampling

No power calculation was conducted to determine sample size due to the qualitative design. Interviews were conducted until saturation was reached and no new information was emerging from the interviews [15]. We determined when saturation was reached in discussion between the local research team and the COMGAP-S Principal Investigator who has extensive experience in qualitative data collection and analysis. This was a non-random convenience sample. Potential participants were contacted through a contact list provided by the video production manager, a Community Physician from the University of Jaffna, who oversaw the production of the video clips. The Community Physician provided a list of people involved in creating translated scripts, directors, university students from drama and medical programmes, and a consultant psychiatrist. While 34 people participated in the adaptation and re-filming of the videos, only 17 provided their names to the Community Physician. Out of these, 12 people consented to participate in the in-depth interviews. Participant characteristics are presented in Table 1 below. Eight participants were male, four were female. Eleven participants signed written consent forms, and one participant gave oral consent over the phone. Participants included four medical students, two doctors, three university lecturers, one government worker, and one self-employed person. These participants filled the following roles in the video production: coordination, director, script writer, and video production/camera operator.

**Table 1:** Participant characteristics

Participant	Occupation	Role in the study	Language of interview	Gender
VP/84	Doctor	Coordination	English	Male
VP/51	Lecturer- Drama	Director	Tamil	Female
VP/02	Lecturer	Director	Tamil	Female
VP/68	Medical Student	Script Writers	English	Female
VP/55	Doctor	Coordination	English	Male
VP/18	Medical Student	Script Writers	Tamil	Male
VP/17	Medical Student	Script Writers	Tamil	Male
VP/61	Medical Student	Script Writers	Tamil	Female
VP/56	Medical Student	Script Writers	Tamil	Male
VP/93	Lecturer	Director	Tamil	Male
VP/22	Self Employed	Director	Tamil	Male
VP/75	DS Office	Video production/ cameraman	Tamil	Male

### Data collection

Participants were given Participant Information Sheets and Consent Forms in local Tamil language and provided informed consent before the start of the interview. Interviews took place in a private room at the University of Jaffna at a time convenient for the participant. The interviewer was a member of the COMGAP-S team with experience in qualitative interviewing and used an open-ended topic guide. This topic guide was created by members of the COMGAP-S team who have extensive experience in qualitative data collection and analysis. The topic guide was reviewed and revised as necessary by members of the local research team at THEME Institute before interviews commenced. Interviews were audio recorded and conducted in either English or Tamil, depending on the preference of the participant. All interviews conducted in Tamil were translated into English and the audio recordings were transcribed by the interviewer verbatim as word documents.

### Adaption of videos

*Mental Health in the Tamil Community* was used to understand and implement local idioms of mental health and distress [16]. The decision made to film in multiple locations such as busy hospitals and small primary care clinics was to portray the settings as a reflection of the local context.

### Validation of scripts

The original mhGAP videos were transcribed into English and sent to the local research team in Sri Lanka. Initial translation to Tamil from English, after which the Tamil translation was reviewed by a group of local experts in Sri Lanka including a psychiatrist, community physician and a dramatist. After this was completed, the translated scripts were circulated amongst a steering group of local experts within Sri Lanka for final approval.

### Data analysis

The authors used an inductive approach to the qualitative data analysis and no specific codes or analytic categories were pre-determined; all codes emerged from the gathered data. Three independent coders read interview data to look for key themes, and any discrepancies were resolved through group discussion until the final set of codes was created. Thematic analysis was used to describe implicit and explicit ideas from within the interview data set and codes were developed to represent identified themes that emerged from the data [17].

## Results

Through the iterative coding process and discussions between coders, the following themes emerged: overall assessment, translation, language and mental health, challenges/barriers, interdisciplinary cooperation, coordination, conflict, visualisation importance, validation, expectations, opportunity, knowledge gained, and recommendations. The themes, as well as the five general concepts which cover them, are shown in Table 2. Emergent themes will be discussed in detail in this section and summed up by general concept in the discussion section. The words of the participants are quoted verbatim from the transcripts or translations, with clarifications and contractions in brackets where necessary.

**Table 2:** Emergent themes categorised under general concepts

General Concept	Emergent Themes
Overall perception	Perception of process
	Expectations
	Opportunity
	Recommendations
Technical issues	Translation
	Language and mental health
	Validation
Knowledge	Importance of visualisation
	Knowledge gain
Barriers	Constraints
	Conflict
Working as a team	Interdisciplinary cooperation
	Coordination

### Perception of process

The majority of participants perceived the process as positive and expressed appreciation and pride regarding the experience of re-filming the videos. One participant commented:“I think this is good learning for me and the team and then also we have learnt a lot of things by involving in this and also we are proud that we were able to produce this video with all the limited facilities” (VP84). Another participant noted: “Truthfully the video has turned out very well [...]This was really a good experience” (VP93).

### Translation

Translation was one of the richest and most readily apparent themes to emerge from the interviews, possibly as many of the participants were directly involved in script adaptation. Tamil is one of the official languages of Sri Lanka, but in the words of one participant: “[T] he Tamil differs from place to place, hence we would have to think about all the people in Sri Lanka as well” (VP17). To clarify, the COMGAP-S study includes the Northern Province only, therefore local speakers from the region were engaged to carry out the translations. The difficulties that participants related include adapting the script from written Tamil to spoken Tamil: “Not everyone can understand [written Tamil]”, (VP17). Another issue involved aligning the scripts to the speech patterns used in the Northern Province. Responses from participants involved in the translation indicate that local experts from the region perceived the importance of active involvement in translating and adapting the training materials. This involvement was important not only for the purposes of ensuring the best localised cultural fit, but also to find the proper equivalents of phrases or words that were difficult to translate in the origin language. As expressed by one participant: “[I] dioms and phrases, which were common in the English language, [...] we had to tweak and make it user friendly for the doctors of our culture” (VP68). Appropriate inclusion of local expertise in linguistic and medical matters of translation was identified by participants as an important contribution which helped remove obstacles in the adaptation process: “Since I am from around here, the language pattern is not a problem” (VP02).

### Language and mental health

The way language intersected with the conceptualisation of mental health emerged as a theme from participants, who noted Tamil language has different terminology and understanding surrounding discussion of mental health issues as compared to English: “[S] ome terminology that is used in English is acceptable in their culture. But when you try to translate these terminologies to Tamil some problems arise” (VP56). Cultural appropriateness of certain mental health questions in the English-language version of the script were understood as too direct for inquiry of the patient: “sometimes [...] in our setting we can’t ask the health seeker some of these things directly” (VP55);“If a doctor asks a patient “Are you mentally all right?“ the context is correct in English, but when translated to Tamil it would become awkward for the doctor to ask such a thing, so the cultural difference was a main problem” (VP56).

Cultural adaptation appeared particularly salient to participants when adapting the scripts: “[D] irect language translation is not involved. The details must be translated to fit our culture here.” (VP56). It also appeared important to ensure the relationship between doctor and patient within the Sri Lankan context was modelled well: “When we were in first year we understood how a doctor would speak to a patient if they come to a clinic, how they would present themselves, how a patient would explain their disease” (VP17). Participants also noted it was vital to ensure this relationship between doctor and patient within particular settings was modelled appropriately: “[A] lso we had to put it in a way that is appropriate to use in a hospital setting by a doctor” (VP68).

### Constraints

Participants noted resource and budgetary constraints acted as barriers to adaptation. Several participants pointed out that filming the videos was an involved process and the time allocated was not sufficient, thus they appeared to feel constrained by available resources. The majority of the filming was done on location and due to the busyness of clinic settings, sometimes filming had to be rescheduled for the weekend only. Further, due to the remoteness of certain clinics, transportation also presented a problem for the participants. Budgetary constraints were also noted as a challenge and participants commented that this included camera quality, which was perceived as resulting in lower video quality. Further, participants stated that some work had to be carried out on a volunteer basis due to a lack of funding. Additionally, participants noted they wished they had been better informed prior to the adaptation work: “It is a very useful concept indeed, yet we would have been able to do more if the proper knowledge and equipment were provided” (VP61).

### Interdisciplinary cooperation

Interdisciplinary cooperation was understood as an important component of developing and filming the videos and appeared to be understood as a positive experience. One participant noted: “ … working with people of different disciplines, from doctors, consultant psychiatrists and veteran dramatists; that’s from one side and from the other side we also got to work with directors, cameramen, technicians and all the artists and the faculty allowed kids to participate there so that was one kind of experience, to be able to work with them” (VP68).

### Coordination

Coordination emerged as a particularly relevant theme, and participants noted difficulties in organizing participants from such disparate fields. Due to various shooting locations and timing issues, participants stated that gathering actors, technical crew and supervisors together was a challenge. Participants commented on how diverse perspectives and understandings of how to coordinate and work as a team made the experience difficult: “[T] hat was kind of a challenge because we got directives from different sides” (VP68). However, most participants highlighted that the process was an important learning opportunity and generally appeared to view the experience as positive. There were however conflicting views on who should be responsible for overall organization, with some participants noting they thought the film directors for each video clip should be in charge, while others appeared to prefer overall supervision from one person only.

### Conflict

One theme which emerged clearly was around perceived conflict between those involved in filming/acting and medical professionals/students regarding expectations around creative vision. Medical professionals noted they wanted the videos to closely mimic clinical practice, however they appeared to feel that their vision differed from the filming/acting crew: “[T] hey wanted to do it in an artistic way but we wanted to do it in a natural way” (VP18); “[I] n some cases we had to be strict and say no, this is not how this is projected” (VP68). The filming and acting professionals appeared to place more importance on employing visual tropes and symbols to convey messages: “[W] hen the child’s mother cries if the doctor touches her hand only the humanity will be revealed, which means that the doctor is showing that he is there for them. Although the medical students did not like this idea...” (VP93). Some participants reported trying not to upset the filming and acting team for fear of escalating tension and conflicts. Younger participants, such as the medical students, noted they often found themselves serving as coordinators and mediators between the two sides of the conflict and felt this was a difficult experience for them.

### Importance of visualisation

Participants noted they perceived the experience of moving from a written script to a video as positive as it seemed to bring the material to life. Participants noted the videos were important as training aids and were valuable in that they showcased what mental health disorders looked like and provided examples for how patients may present in a hospital setting: “We can just read a textbook [...] but when we see the same thing visually [...] the way it stays with us will be higher.” (VP56). Medical students, who are expected to absorb great quantities of information in their education, agreed that “[s] eeing information about particular diseases in a video was better in comparison to reading it from a book” (VP61).

### Validation

Psychiatric consultants and community physicians from the region implemented steps to validate the scripts before shooting. Translation followed the usual steps for psychological tools and instruments, with translation, back translation, and independent verification by at least two subject experts (community physician and consultant psychiatrist). Participants noted this process was vital to document so others would have the ability to re-film the videos in other contexts and languages in the future: “[W] e thought that we should make this methodology to be available for whoever want to reproduce in their own language, native language, when they want” (VP84).

### Expectations

Several of the participants expressed expectations for the videos produced “to be studied and that to be available for other people in the world” (VP84) and appeared to perceive the process of adaptation as a model for others to follow. Participants also expressed an expectation that the videos would be used for education of the general public on mental health: “The psychiatry doctors know that this video provides the opportunity to educate the general population” (VP61). Further expectations emerged, including disseminating the video clips to other parts of Sri Lanka, translating the videos into Sinhalese (the other official language of Sri Lanka), and the possibility of uploading the videos to a public sharing platform such as YouTube.

### Opportunity

Medical professionals and filming and acting teams noted they felt the experience of re-filming the videos as an opportunity and listed several positives coming from the experience including enhanced knowledge for the medical students, technical experience for the filming crew, and greater exposure for the drama students. One participant noted:“We do not get opportunities like this here. I think the fact that we got this opportunity is a good thing. More than myself, my fellow students getting the opportunity is what I feel is a good thing” (VP93).

### Knowledge gain

Participants noted they gained knowledge during the video production, particularly specialist knowledge centred on psychiatry and clinical presentation of mental health disorders. This was a particularly salient component for the medical students as they were able to obtain new knowledge on psychiatric disorders and how they may present in the region:“We obtained new knowledge about psychiatry in taking part in this. This community is seeing psychological diseases. We got information as to how these diseases were” (VP61). Other participants noted they felt knowledge gain extended beyond them to other stakeholders in the region who could find the videos valuable: “In the national ministry and the Provincial Ministry of Health are very keen to use those videos. I’m sure this will be useful for them” (VP84).

### Recommendations

On the question of what they would recommend for future similar activities, participants had multiple suggestions. Better preparation was among the most frequent recommendations and included providing more information, giving actors the opportunity to rehearse, and planning funding more appropriately to ensure a high quality output. Several participants expressed the opinion that medical professionals as actors would improve the accuracy of clinical representations: “If the director and actor were in the medical field it would have been better” (VP18). Others appeared to understand the utility of the video format beyond the particular mental health driven project: “It would be great if there are videos for mental health related or any other health condition related issues. These resources would be useful for the society” (VP56).

The need for quality control and regular feedback while producing the videos was also highlighted by participants in order to ensure some level of standards are maintained: “Several rehearsals were done and we all [ ….] were watching that and we were giving feedback for them to improve that and so we tried to maintain standards in each and every steps of this video” (VP84).

Adhering to already existing scripts was described as a limitation by some participants, who saw it as lack of flexibility in adapting the videos to local context: “A limitation is that the lines that were given to us were very firm. So we got know some details that if we do it this way it would be better. But we can say that the flexibility was less in the rigid firm work we got” (VP56). Participants stated a preference for building new scripts based on existing scenarios, which would be more faithful to the ways of expressing mental health suffering and to the medical methods employed by healthcare professionals: “ … .we were not asked to write something new, it was already been filmed; there was a set line there. A line of continuity, where it should start and where to end, it was all there. But the local hospital setting and health care system is slightly different, so we had a problem in that” (VP55).

## Discussion

Cultural adaptation of instruments used for mental health intervention has growing importance in the light of increased sensitivity for intercultural differences. Frameworks have paid attention to the details of the concepts of distress, treatment components and treatment delivery [18]. The themes which emerged from this study were united under five general concepts: overall perception of process, technical issues, knowledge, barriers, and working as a team.

The perception of the process of re-filming the videos appeared to be positive by the majority of the participants who expressed a sense of appreciation and pride in the process and the outcome. This suggests a sense of ownership was conferred by the direct involvement of participants in the adaptation of scripts and the filming of WHO mhGAP videos. Participants also noted they had expectations about the future use of the videos, suggesting they should be made available to the general public (both locally and globally) as they felt they had created a model of best practice for others to follow. Locally-driven creation and adaptation of materials related to the mental health of the community is an important point of international collaborations, given the history of institutions or researchers from high-income countries pushing for their own agenda [19].

### Technical issues

Technical issues noted in interviews related to translation, language and mental health. Translation from written Tamil to spoken Tamil was considered difficult as the spoken language is considered by speakers to diverge significantly from the literary written language. Marked regional variations in dialect within Sri Lanka also complicated translation. The team used best practices to circumvent this by including local experts who live and work in the Northern Province region, such as the consultant community physician, consultant psychiatrist, and a dramatist for the current project. Interviewees noted that adaptation of the scripts presented occasional difficulties as some phrases or manner of speaking did not exist in English or were not appropriate to use in Tamil settings - a problem much discussed in the global mental health community [20]. The “gold standard” use of translation, back translation, and then verification by two independent local experts was acknowledged as necessary by interviewed participants. A good rule of thumb for future projects related to mhGAP training therefore is to ensure close linguistic correspondence, similar to the procedure employed for localization and adaptation of instruments [21]. This ensures material produced will be useful and provide familiar examples of exchanges that trainees may encounter in their local settings.

Closely related to issues of translation was the intersection of language and conceptualisation of mental health in the Tamil community. The Tamil community in Northern Province has a certain understanding of mental health and these views had to be taken into account in the adaptation [19]. For example, “thinking too much (*yosanai*)” is a traditional explanatory belief for people who are experiencing psychosocial problems in the Tamil community [22].

Further, interactions between health care workers and patients are understood in particular ways in the Tamil community. In the original mhGAP videos there are examples of how a doctor should ask questions about mental health which were deemed by the adaptation team as inappropriate in the Tamil setting. This needed to be respected accordingly so video training materials could provide vignettes acceptable both to the mhGAP trainees and potential patients. Additionally, due to the manner in which clinical posting of medical professionals are organised in Sri Lanka, mhGAP trainees often came originally from all over the country. The training materials thus served the double purpose of modelling clinicians’ behaviour in mental health diagnosis and indirectly educating them about the cultural norms of communication in the Northern Province. Our experience underscored that in adaptation, it is important to interrogate the source material and check how it relates to the groups affected during roll-out. While this is understood as an integral part of the process of cultural adaptation of novel tools and manuals, project teams need to keep these actions in mind when handling pre-existing or toolkit materials such as the mhGAP-IG. Our findings further imply that good adaptation and training can confer unexpected benefits, which can support the argument for wider implementation and additional uses of the produced material.

### Knowledge

Visualising information on mental health that is often only presented in written form was considered by participants as a valuable contribution to increasing knowledge, a learning tool and a potential medium for other health related conditions. The ability to see and hear how patients within the Tamil community may present with mental health disorders was viewed as bringing information to life that was previously inaccessible and provided a more comprehensive understanding. This is in line with pedagogic research which asserts that learning materials should focus on task-focused instructional practice which emphasise learning, enjoyment and mastery [23].

Clinical vignettes would be especially important in regions similar to Northern Sri Lanka where medical schooling may incorporate abbreviated or didactic teaching on mental health disorders without a practical or observation component. Additionally, the theme of knowledge gained was seen as both knowledge gained for oneself (increased individual understanding) and knowledge explicated for others such as Ministry of Health. Participants noted that it was often difficult to explain the importance of mental health to Ministry of Health officials and found the videos to be useful tools that could be easily understood by non-experts. The role of clinical vignettes to reduce stigma and enhance understanding among non-specialists has been noted in research [24]. The Northern Ministry of Health has also expressed interest in using the videos in future as learning tools, pointing to the flexibility of the video format, which can extend its utility far beyond the specific research project. The adapted videos are already being utilised within medical student exams at the Jaffna Teaching Hospital, Northern Province, where the local consultant psychiatrist felt they were a useful pedagogical tool to impart information. This indicates that the lifetime of this could be useful in multiple contexts. Such possibilities need to be considered at the outset of adaptation, in order to create materials that can serve secondary audiences if necessary and to establish conditions and possible restrictions to dissemination.

### Barriers

Participants in the study were able to freely discuss barriers that challenged their ability to complete the re-filming project successfully. Logistic issues and internal conflict in the adaptation team took the foreground. The timeline allocated for the re-filming was dependent on the overall project time and participants noted it was insufficient. To achieve realistic conditions, filming was completed in clinic settings, but this had to be scheduled around patient care, often on weekends, which resulted in participants having to allocate extra time to the project. Many of these shooting locations were located in remote areas, meaning that transportation to and from the clinics impacted on participants’ time. While unforeseen circumstances can arise during filming, future initiatives of the kind would need to give due consideration beforehand to logistics and feasibility of the planned schedule to reduce discrepancies and accommodate all involved. Budgetary constraints also emerged as an issue as participants felt insufficient funds had been allocated for the re-filming. The budget was initially developed in collaboration with local stakeholders; however, due to the interest and plans made by the adaptation team and consultants, costs soon ballooned outside of budgetary limits. These findings serve to demonstrate that technical crew and clinical teams on such projects may have diverging expectations and priorities. Considering the often limited funding for mental health research in LMIC, planning for budget and time allocation needs to move beyond the primary concerns of clinical teams of content and veracity and engage the technical crew in order to be realistic [25].

The difference of priorities between those involved in acting/filming and the medical professionals brought about internal conflict in the team. The acting/filming team seemed to want the films to feel artistic and reflect visual tropes, while the medical professionals placed more importance on ensuring clinical practice was reflected appropriately. At times, the two groups felt they were at cross-purposes, while some of the medical student participants felt they were placed in an impossible position trying to mediate conflict. As noted below, this could be mediated in future by appointing a senior person to act as general manager and mediator between differing agendas. Having clear responsibility for final decision-making may serve to reduce conflict, balance the viewpoints of diverse professionals and avoid shifting the mediating role.

### Working as a team

Closely related to the issue of conflict were emergent themes centred around ability to work as a team. As re-filming required cooperation between multiple disciplines ranging from physicians to psychiatrists to adult and child actors to medical students, cooperation and coordination between people was often challenging. This was further complicated by differing understandings of teamwork and role delineation. While the COMGAP-S project had a field coordinator, this was not sufficient to bring together all the stakeholders into a team. Participants expressed conflicting views on how this could be solved in future, with some remarking that individual film directors should be responsible for production of their own clips and others stating an overall supervisor for all filming would be more appropriate. It was important to the Principal Investigator of the project to preserve the autonomy of the local partners in decision-making, while respecting schedules; the message received from this collaboration wasthat expectations need to be clearly discussed on all sides when starting out on such initiatives.

Participants’ experiences prompted them to give a number of recommendations for similar future initiatives. One such recommendation was that more time should be given to prepare and rehearse, indicating they felt overwhelmed at times by the influx of information provided. Future projects which plan to re-film mhGAP videos in local context should consider including space for mental health awareness raising activities with the team prior to filming to ensure all participants feel prepared and comfortable with the material.

### Limitations

The current study is limited by its small sample and bias of responders. Because only willing responders were interviewed after the fact, it is possible that certain experiences may have gone unrecorded, especially negative experiences that may have held back potential participants, leading to self-selection bias. Additionally, recall bias may have operated given that participants were interviewed 1 year after concluding the process of video production. Being interviewed by members of the research team (which did not take part in or influence the video adaptation, but did disburse the budget) may have influenced participants to give answers which skew to the socially desirable, given that the goals of the research study are consistent with good-quality adaptation, and to issues of organization and budgeting due to perceived management/oversight role of the research team.

## Conclusion

This project aimed to understand the experiences of those who were involved in the adaptation and re-filming of the WHO mhGAP video training materials for local context in Northern Province, Sri Lanka. To our knowledge, this is the first study to explore adaptation of the mhGAP video materials, which are reportedly used in over 90 countries to facilitate mental health knowledge for non-specialists [26]. We have added to the knowledge base on locally-based trainings on conducting cultural and language adaptations, and our findings indicate participants felt adapting the mhGAP films to local context was vital to ensuring training materials were culturally appropriate and valid. Frequently studies incorporating localised materials, especially video, do not go into detail regarding the process of adaptation, with the consequence that there is not much information on what changes have been carried out and how decisions were made. We have attempted to bridge that gap and give prominence to some of the considerations which researchers need to take into account when planning the schedule, budget and logistics around adaptation, as well as showing the inner workings of content and form alterations. This project demonstrates that locally adapted mhGAP material can not only facilitate more culturally appropriate and relevant training material, but also increases sense of ownership and engagement with the research process, promising possibilities of further dissemination - essential prerequisites for ensuring success and sustainability of mhGAP implementation.

## Data Availability

Transcripts, translations and audio recordings of the interviews are available from the Primary Investigator of the COMGAP-S project Dr. Shannon Doherty, who is also corresponding author for this manuscript. Given that identification of participants is possible from the data, they will not be shared publicly. Interested persons may inquire through the contact details of the corresponding author.

## Abbreviations

CDC: Centers for Disease Control and Prevention
IMC: International Medical Corps
mhGAP: Mental Health GAP Action Program
WHO: World Health Organisation
